# The Rinderpest Poison

**Published:** 1866-09

**Authors:** 


### The Rinderpest Poison.

In the report made by Mr. Crookes, F. R. S. to Her Majesty's Commissioners upon the subject of the application of disinfectants in arresting the spread of the cattle plague, the following conclusions are drawn:—(1.) The disease is eminently infectious. (2.) The infecting matter is neither a gas nor a volatile fluid. (3.) The specific disease-producing particles are organized, and possess vitality. (4.) The virus of cattle plague is most probably a body similar to vaccine lymph, and consists of "germinal matter," having a physiological individuality. (5.) The blood poisoning set up may legitimately be called fermentation; "it is a decomposition caused by the act of nutrition in the living cell," the result of which is the production of an infinite number of similar bodies. (6.) It is not certain that the multiplication of these cells is the *immediate* cause of the blood-poisoning. (7.) The ordinary notion, that the virus of contagion consists of decomposing organic matter which is in transition from a more complex to a simple chemical constitution is plausible, but does not meet some of the facts. If it were accepted it would fail to account for the rapid and extensive multiplication of the virus-germs. (8.) The germs may be carried to a limited extent through the air, especially through the medium of fogs, but they do not retain their vitality for any length of time in the atmosphere. (9.) There is no evidence to show that the germs can propagate themselves apart from the animal. *Lancet.*

